# Paracrine cyclooxygenase-2 activity by macrophages drives colorectal adenoma progression in the *Apc*^*Min/*+^ mouse model of intestinal tumorigenesis

**DOI:** 10.1038/s41598-017-06253-5

**Published:** 2017-07-20

**Authors:** Mark A. Hull, Richard J. Cuthbert, C. W. Stanley Ko, Daniel J. Scott, Elizabeth J. Cartwright, Gillian Hawcroft, Sarah L. Perry, Nicola Ingram, Ian M. Carr, Alexander F. Markham, Constanze Bonifer, P. Louise Coletta

**Affiliations:** 1 Section of Molecular Gastroenterology, Leeds Institute of Biomedical & Clinical Sciences, University of Leeds, St James’s University Hospital, Leeds, LS9 7TF United Kingdom; 2 Section of Translational Medicine, Leeds Institute of Biomedical & Clinical Sciences, University of Leeds, St James’s University Hospital, Leeds, LS9 7TF United Kingdom; 3 Section of Experimental Haematology, Leeds Institute of Cancer and Pathology, University of Leeds, St James’s University Hospital, Leeds, LS9 7TF United Kingdom

## Abstract

Genetic deletion or pharmacological inhibition of cyclooxygenase (COX)-2 abrogates intestinal adenoma development at early stages of colorectal carcinogenesis. COX-2 is localised to stromal cells (predominantly macrophages) in human and mouse intestinal adenomas. Therefore, we tested the hypothesis that paracrine Cox-2-mediated signalling from macrophages drives adenoma growth and progression *in vivo* in the *Apc* ^*Min/*+^ mouse model of intestinal tumorigenesis. Using a transgenic C57Bl/6 mouse model of *Cox-2* over-expression driven by the chicken lysozyme locus (*cLys-Cox-2*), which directs integration site-independent, copy number-dependent transgene expression restricted to macrophages, we demonstrated that stromal macrophage Cox-2 in colorectal (but not small intestinal) adenomas from *cLys-Cox-2* x *Apc* ^*Min/*+^ mice was associated with significantly increased tumour size (P = 0.025) and multiplicity (P = 0.025), compared with control *Apc* ^*Min/*+^ mice. Transgenic macrophage Cox-2 expression was associated with increased dysplasia, epithelial cell Cox-2 expression and submucosal tumour invasion, as well as increased nuclear β-catenin translocation in dysplastic epithelial cells. *In vitro* studies confirmed that paracrine macrophage Cox-2 signalling drives catenin-related transcription in intestinal epithelial cells. Paracrine macrophage Cox-2 activity drives growth and progression of *Apc* ^*Min/*+^ mouse colonic adenomas, linked to increased epithelial cell β-catenin dysregulation. Stromal cell (macrophage) gene regulation and signalling represent valid targets for chemoprevention of colorectal cancer.

## Introduction

There is incontrovertible genetic and pharmacological evidence from rodent models that the inducible isoform of prostaglandin (PG) G/H synthase (PTGS2), also known as cyclooxygenase (COX), COX-2 plays an important role in the early stages of intestinal tumorigenesis during adenoma (or polyp) development^1–3^. These pre-clinical studies led to investigation of the chemopreventative efficacy of selective COX-2 inhibitors in randomized, placebo-controlled colorectal polyp prevention trials in humans^4^. Predictably, both celecoxib and rofecoxib use were demonstrated to be associated with a significant reduction in polyp recurrence at colonoscopy, compatible with a future role for selective COX-2 inhibitors in colorectal cancer (CRC) chemoprevention^5–7^. However, long-term use of coxibs caused an excess of cardiovascular thrombotic events such as myocardial infarction^8^, limiting future use of this class of drugs for primary prevention of ‘sporadic’ CRC. Therefore, there is a requirement for novel approaches, which harness the clear anti-neoplastic efficacy of COX-2 inhibition, whilst minimizing or avoiding systemic toxicity.

We, and others, have described that COX-2 is expressed predominantly by stromal cells in human and rodent intestinal adenomas^9–15^, rather than the dysplastic epithelial cells that eventually undergo malignant transformation. Several cell types are believed to contribute to the stromal COX-2-positive cell population in human and mouse intestinal adenomas including macrophages^9–11, 15^, (myo)fibroblasts^13, 14^ and endothelial cells^12^. In human tumours, the predominant COX-2-positive stromal cell population is the CD68-positive macrophage^10, 11^.

The predominant stromal cell localization of COX-2 in adenomas implies a role for paracrine Cox-2-mediated signalling during the early stages of intestinal tumorigenesis. We have demonstrated that mouse macrophage Cox-2 induces tumorigenic behaviour (including anchorage-independent growth and resistance to apoptosis) of non-transformed IEC-6 rat intestinal epithelial cells *in vitro* ^16^. More recently, others have provided evidence that paracrine COX-2-mediated signalling from stromal fibroblasts can drive proliferation of human CRC cells *in vitro* ^17^.

The role of paracrine COX-2-mediated signalling from stromal macrophages during the early stages of intestinal tumorigenesis *in vivo* remains unclear. The *Apc* ^*Min/*+^ mouse model of the early stages of intestinal tumorigenesis remains the predominant rodent model of ‘sporadic’ colorectal adenoma development for investigating chemoprevention strategies^18, 19^. Despite the fact that the majority of adenomas are present in the small intestine (SI) and newer conditional mouse models, effectiveness of chemopreventive agents in the *Apc* ^*Min/*+^ mouse model is highly predictive of clinical efficacy^4–7^. Therefore, we tested the hypothesis that increased expression of Cox-2 by macrophages promotes *Apc* ^*Min/*+^ mouse intestinal tumorigenesis using a transgenic model of macrophage-specific Cox-2 over-expression.

## Results

### The c*Lys-Cox-2* transgenic mouse model of macrophage-specific Cox-2 expression

**Figure 1:**
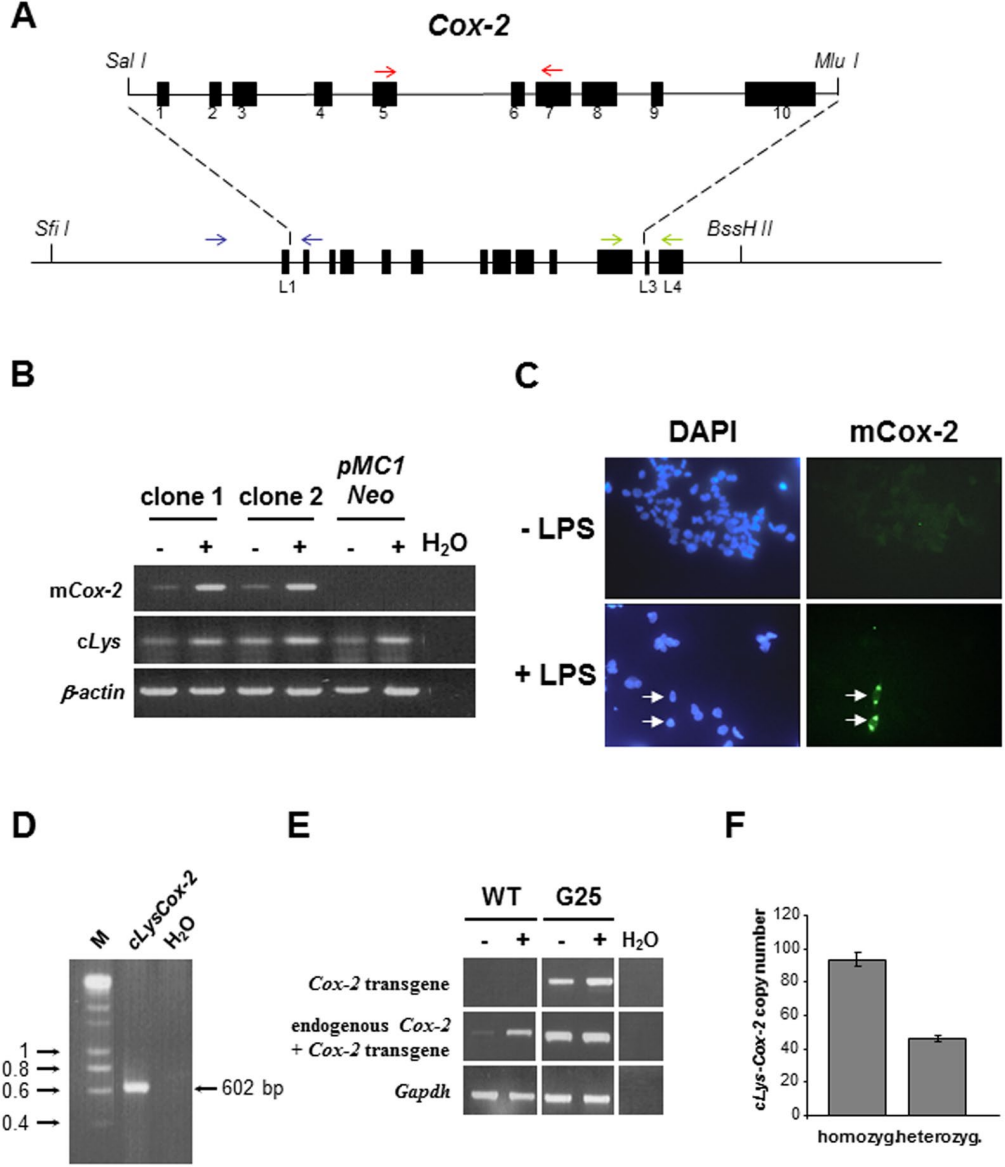
Generation of *cLys-Cox-2* mice. (**A**) Insertion of a complete mouse *Cox-2* genomic DNA clone consisting of 10 exons (numbered solid boxes), as well as 68 bp of 5′-UTR and 239 bp 3′-UTR sequence, into plasmid *pIIIiLysV*_*Sal30*_ flanked by exon 1 (5′) and exon 3 (3′) of *cLys* (labelled L1, L3). Linearisation of plasmid DNA (termed *cLys-Cox-2*) was carried out using *Sfi I* and *BssH II* restriction enzymes. Arrow pairs denote PCR primers (see Supplementary Table 1) specific for endogenous and transgenic mouse Cox-2 mRNA (mCox-2 cDNA; red), transgenic Cox-2 mRNA only (cLys-Cox-2 cDNA; green) and transgenic DNA (cLys-Cox-2 gDNA; blue). (**B**) RT-PCR for mouse *Cox-2* mRNA (red primer pair), chicken *lysozyme* (c-*Lys*) mRNA and chicken *β-actin* in stably transfected HD11 cell clones, which were either unstimulated (−) or activated (^+^) with 5 μg/ml LPS for 16 hours. Two clones co-transfected with *cLys-Cox-2* and *pMC1 Neo* both demonstrated inducible expression of mouse Cox-2 mRNA unlike control cells transfected with pMC1 Neo alone. By contrast, all cell clones exhibited inducible endogenous c-*Lys* and constitutive *β-actin* expression. H_2_0 denotes PCR reactions with no cDNA template added. (**C**) Immunofluorescence for mCox-2 protein on unstimulated and LPS-stimulated HD11 clone 2 cells. Left-hand panels show DAPI-stained nuclei only with corresponding FITC-labelling of mCox-2 in 5-10% of LPS-stimulated cells (arrows). Cox-2 protein was not detected in LPS-stimulated *pMC1 Neo*-transfected cells using polyclonal anti-Cox-2 antibody (Cayman 160126; data not shown). (**D**) Identification of transgenic founder animals by PCR genotyping of tail-tip DNA using cLys-Cox-2 gDNA primers (see Supplementary Table 1). (**E**) *cLys-Cox-2* expression *in vivo*. Transcript analysis in BMDMs from G25 transgenic mice and wild-type (WT) controls for transgenic and endogenous *Cox-2* and *Gapdh*. H_2_0 denotes PCR reactions with no cDNA template added. (**F**) Real-time PCR genotyping for the *cLys-Cox-2* transgene in the G25 line. The transgene copy number was determined as the ratio of *cLys-Cox-2*/*Cox-1* DNA divided by two and then subtracted by two (the endogenous *Cox-2* gene copies). Bars represent the mean and standard error of the mean (SEM) values from tail-tip DNA from 30 (homozygous) and 50 (heterozygous) mice. Figure 1B,D and E are cropped gel images. No bands have been omitted by editing.

The chicken *lysozyme* (c*Lys*) gene locus directs copy number-dependent, integration site-independent, macrophage-specific transgene expression in mice^20–22^. We cloned a 5.8 kb mouse genomic *Cox-2* clone consisting of all 10 exons and a 239 bp 3′-UTR fragment into the *pIIIiLysV*_*Sal30*_ vector (Fig. 1A)^20^. Functionality of the linearised 18 kb *Sfi I*-*BssH II* DNA fragment (including 11.5 kb of the cLys 5′-flanking region, which contains all the *cis*-regulatory elements necessary for macrophage-specific expression in mice^23^, but excluding the majority of the adjacent chicken *Gas41* gene at the 3′-end of the *cLys* locus^24^), was tested in stably transfected chicken HD11 macrophages. Inducible mouse *Cox-2* expression was demonstrated in two independent stably transfected HD11 cell clones (Fig. 1B,C). The 18 kb vector was then injected into the pronucleus of fertilised oocytes to generate transgenic founder C57Bl/6 (B6) x CBA animals, which were screened by transgene-specific *cLys-Cox-2* PCR (Fig. 1D). Three founders termed G5, G25 and G26 were identified from 26 offspring from 5 foster mothers. Only G5 and G25 founders transmitted the transgene at low frequency in four (9/34) and three litters (4/27), respectively. In order to test transgenic expression *in vivo*, we measured *cLys-Cox-2* transcript levels in bone marrow-derived macrophages (BMDMs). Unlike G5 BMDMs, unstimulated BMDMs from the G25 line exhibited *cLys-Cox-2* mRNA expression, which was induced further by (LPS) and γ-interferon (IFN), in parallel with endogenous mouse *Cox-2* expression (Fig. 1E). Therefore, G25 mice were used exclusively for further experiments (termed *cLys-Cox-2*) and were bred to near homozygosity on the B6 background (N6 generation backcross) using an in-house real-time PCR method for transgene genotyping (Fig. 1F). The hemizygous transgene copy number was determined as 47 by direct comparison with endogenous *Cox-1* genomic DNA (Fig. 1F). Whole genome DNA sequencing of a homozygous B6 G25 *cLys-Cox-2* mouse revealed that there were 5 possible transgene insertion sites, four of which were flanked by major satellite (MaSat) repeats in large, transcriptionally silent intergenic regions^25^, and one of which was located in the centre of the first (360 kb) intron of the *MAM domain containing glycosylphosphatidylinositol anchor 2* gene.

**Figure 2:**
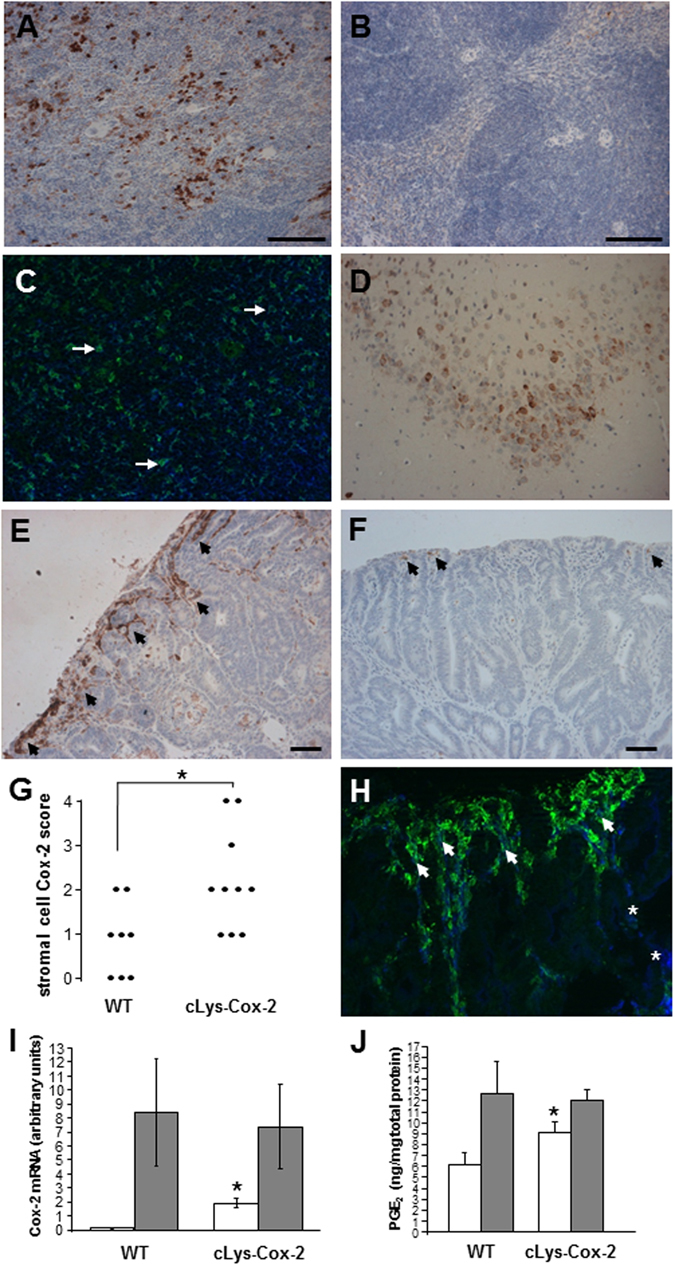
Cox-2 expression in *cLys-Cox-2* mouse tissues. Cox-2 immunohistochemistry on (**A**) spleen from a *cLys-Cox-2* mouse (day 100) showing Cox-2 protein localisation to transgenic splenocytes unlike (**B**) spleen from an aged-matched WT mouse. (**C**) Dual immunofluorescence for Cox-2 (green) and F4/80 (blue) demonstrating localisation of Cox-2 to splenic macrophages (arrows show examples). Cox-2-positive macrophages represent a subset of the total F4/80-positive splenocyte population. (**D**) Localisation of Cox-2 protein to hippocampal cells in *cLys-Cox-2* mouse brain. No Cox-2 protein was detectable in equivalent sections from WT brain (data not shown). Cox-2 protein localization in transgenic *cLys-Cox-2* x *Apc* ^*Min/*+^ mouse colonic tumours and non-transgenic *Apc* ^*Min/*+^ mouse colonic adenomas. (**E**) Strong Cox-2 staining (Cox-2 score 4) in superficial stromal cells (arrows) below the luminal surface of a *cLys-Cox-2* x *Apc* ^*Min/*+^ mouse colonic tumour. Size bar = 50 μm. (**F**) Patchy Cox-2-positive superficial stromal cells (arrows) in a non-transgenic *Apc* ^*Min/*+^ mouse adenoma (Cox-2 score 1). Size bar = 50 μm. (**G**) Individual stromal Cox-2 scores of transgenic (n = 10) and non-transgenic (n = 8) *Apc* ^*Min/*+^ mouse tumours. *P = 0.02, Mann-Whitney U test. (**H**) Dual immunofluorescence for Cox-2 (green) and the macrophage marker F4/80 (blue) in a *cLys-Cox-2* x *Apc* ^*Min/*+^ mouse colonic tumour. Greater than 90% of Cox-2-positive stromal cells exhibit F4/80 positivity (closed arrows) but are only a subset of the total intra-tumoral F4/80 macrophage population (asterisks) in transgenic colonic tumours. (**I**) Cox-2 mRNA levels in colonic tumour tissue (shaded columns) and adjacent non-neoplastic colonic mucosa (open columns) from non-transgenic (WT) and transgenic (cLys-Cox-2) animals. Columns and bars represent the mean and SEM from three separate animals. *P = 0.01 for the comparison with non-transgenic *Apc* ^*Min/*+^ mouse colonic mucosa (Student’s t-test). (**J**) Tissue PGE_2_ levels in colonic tumour tissue (shaded columns) and adjacent non-neoplastic colonic mucosa (open columns) from non-transgenic (WT) and transgenic (cLys-Cox-2) animals. Columns and bars represent the mean and SEM from ten separate animals. *P = 0.12 for the comparison with non-transgenic *Apc* ^*Min/*+^ mouse colonic mucosa (Student’s t-test).

Adult B6 *cLys-Cox-2* mice demonstrated no observable phenotypic differences from wild-type (WT) B6 littermates and bred normally. As expected from a previous study of *cLys* transgene expression in mice^23^, transgenic Cox-2 protein was detected by immunohistochemistry in F4/80-positive splenocytes and hippocampal glial cells in *cLys-Cox-2* mice (Fig. 2A–D).

### Macrophage Cox-2 expression promotes *Apc*^*Min/*+^ mouse colonic tumorigenesis

In order to determine the effect of macrophage-specific Cox-2 over-expression on the early stages of intestinal tumorigenesis, we crossed B6 *cLys-Cox-2* mice with our existing B6 *Apc* ^*Min/*+^ mouse colony.

Cox-2 protein is known to be localized predominantly to superficial stromal cells below eroded luminal epithelium in *Apc* ^*Min/*+^ mouse SI and colonic adenomas^9^. This Cox-2-expressing stromal cell population is heterogeneous but does include macrophages^12, 13^. Transgenic *cLys-Cox-2* x *Apc* ^*Min/*+^ mouse colorectal tumours demonstrated an increase in Cox-2 immunoreactivity in the stromal cell compartment below the luminal tumour surface compared with non-transgenic *Apc* ^*Min/*+^ mouse colorectal tumours (P = 0.02, Mann-Whitney U test; Fig. 2E–G). Greater than 90% of Cox-2-positive stromal cells in transgenic tumours were F4/80-positive macrophages present in a similar distribution (predominantly below the luminal surface) to that observed in non-transgenic *Apc* ^*Min/*+^ mouse tumours (Fig. 2H). Although we observed increased expression and function (measured as the tissue prostaglandin [PG] E_2_ content) of *Cox-2* in *Apc* ^*Min/*+^ mouse tumour tissue compared with adjacent non-neoplastic colonic mucosa (Fig. 2I,J), as has previously been reported^26^, we did not detect increased levels of Cox-2 mRNA or PGE_2_ in whole transgenic tumour samples compared with non-transgenic *Apc* ^*Min/*+^ mouse tumours (Fig. 2I,J). The focal nature of Cox-2 expression in *Apc* ^*Min/*+^ mouse tumours likely explains the failure to demonstrate elevated Cox-2 mRNA levels or tissue PGE_2_ levels in whole transgenic *Apc* ^*Min/*+^ mouse tumours compared with non-transgenic tissue (Fig. 2I,J). However, we did observe a significant increase in *Cox-2* transcript levels, as well as a non-significant increase in PGE_2_ content, in non-neoplastic *cLys-Cox-2* x *Apc* ^*Min/*+^ mouse colonic mucosa compared with non-transgenic *Apc* ^*Min/*+^ mouse colonic mucosa (Fig. 2I,J and Supplementary Information).

**Figure 3:**
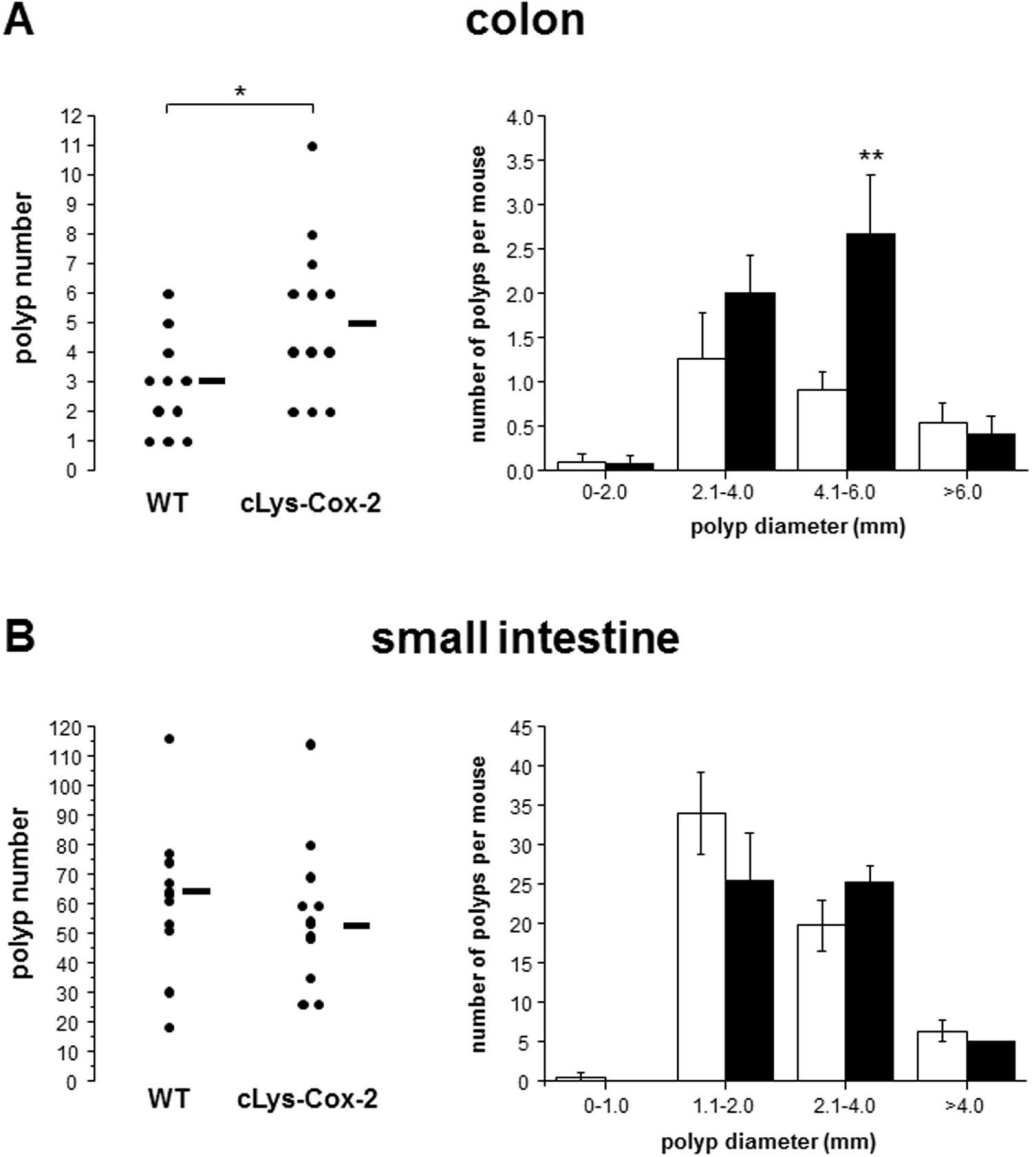
Colonic tumour number and size are increased in *cLys-Cox-2* x *Apc* ^*Min/*+^ mice. Colonic (**A**), but not SI (**B**), tumour multiplicity and size are increased in *cLys-Cox-2* x *Apc* ^*Min/*+^ mice compared with non-transgenic controls. Post-mortem intestinal phenotype analysis was performed at 100-110 days of age by one observer blinded to genotype. Dots represent individual tumour numbers for 11 non-transgenic and 12 transgenic animals. Lines represent median values. Columns and bars represent the mean and SEM number of tumours in different size categories from non-transgenic (empty) and transgenic (shaded) *Apc* ^*Min/*+^ mice. *P ≤ 0.03. **P = 0.025.

**Figure 4:**
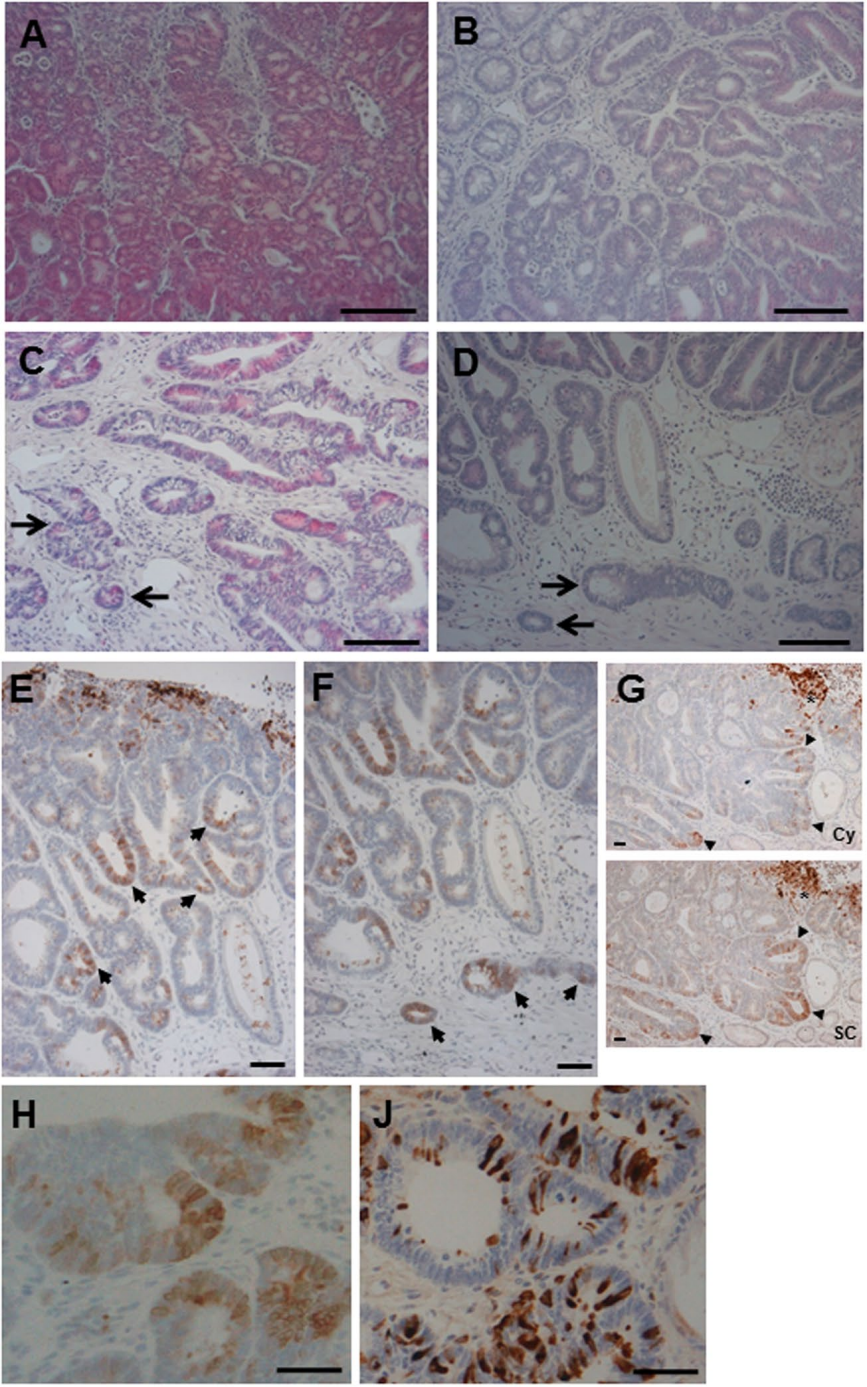
Histological features of *cLys-Cox-2* x *Apc* ^*Min/*+^ mouse colonic tumours stained with H&E. (**A**) *cLys-Cox-2* x *Apc* ^*Min/*+^ mouse colonic tumour demonstrating disorganised clusters of severely dysplastic epithelial cells. Size bar = 50 μm. (**B**) Non-transgenic *Apc* ^*Min/*+^ mouse colonic tumour demonstrating typical features of low-grade dysplasia. Size bar = 50 μm. (**C** and **D**) Separate *cLys-Cox-2* x *Apc* ^*Min/*+^ mouse colonic tumours demonstrating invasion of neighbouring submucosa by dysplastic epithelial cells (arrows). (**E**) Patchy dysplastic epithelial cells staining for Cox-2 (arrows) in a *cLys-Cox-2* x *Apc* ^*Min/*+^ mouse colonic tumour (stromal Cox-2 score 4). Size bar = 50 μm. (**F**) Epithelial cell Cox-2 protein localization in cells at the base of a *cLys-Cox-2* x *Apc* ^*Min/*+^ mouse colonic tumour (arrows). Size bar = 50 μm. (**G**) Adjacent sections of a *cLys-Cox-2* x *Apc* ^*Min/*+^ mouse colonic tumour stained with either the Cayman (Cay) or Santa Cruz (SC) anti-Cox-2 antibody. Note prominent patchy epithelial cell staining (arrowheads) in addition to stromal cell staining (asterisk) in the same distribution in both panels. In general, adenomatous epithelial cell staining with the Santa Cruz antibody was consistently more widespread than with the Cayman antibody. Size bars = 50 μm. Immunohistochemistry for Cox-2 (using the Cayman antibody [**H**]) and lysozyme (**J**) on adjacent sections of a *cLys-Cox-2* x *Apc* ^*Min/*+^ mouse colonic tumour. Size bars = 50 μm. There was no correlation between Cox-2 and lysozyme immunoreactivity in individual adenomatous epithelial cells.

Intestinal phenotype analysis at 100 days of age demonstrated that *cLys-Cox-2* x *Apc* ^*Min/*+^ mice developed a significantly larger number of colonic tumours than non-transgenic *Apc* ^*Min/*+^ animals (P = 0.03, Mann-Whitney U Test; Fig. 3A). Colonic tumours from *cLys-Cox-2* x *Apc* ^*Min/*+^ mice were also larger than in non-transgenic counterparts (P = 0.025, one-way ANOVA) although this was not evident in the largest colonic tumours, perhaps due to the small number of tumours greater than 6 mm in diameter (Fig. 3A). By contrast, there was no significant difference in SI tumour multiplicity or size in *cLys-Cox-2* x *Apc* ^*Min/*+^ mice compared with non-transgenic controls (Fig. 3B). There were no gender-specific differences in tumour size or multiplicity in either *cLys-Cox-2* x *Apc* ^*Min/*+^ mice or non-transgenic controls (data not shown). Consistent with accelerated colorectal tumorigenesis in transgenic animals, 5 of 10 (50%) colonic tumours from *cLys-Cox-2* x *Apc* ^*Min/*+^ mice were adenomas with severe epithelial dysplasia (Fig. 4A,B), whereas 8 colonic adenomas examined from non-transgenic *Apc* ^*Min/*+^ mice contained epithelial cells with only low or moderate grade dysplasia, but no severe dysplasia (P = 0.04, Fisher’s Exact Test). In addition, 3 colonic tumours in *cLys-Cox-2* x *Apc* ^*Min/*+^ mice demonstrated evidence of localized deep invasion of the muscularis mucosae by nests of epithelial cells consistent with tumour progression towards adenocarcinoma (Fig. 4C,D).

Six *cLys-Cox-2* x *Apc* ^*Min/*+^ mouse tumours contained patchy epithelial cells that expressed Cox-2 protein (Fig. 4E,F), in contrast to non-transgenic *Apc* ^*Min/*+^ mouse adenomas, which consisted only of Cox-2-negative epithelium, a finding similar to previous immunohistochemical studies of non-transgenic *Apc* ^*Min/*+^ mouse tumours using the same rabbit polyclonal anti-Cox-2 antibody from Cayman Chemical Co.^9^. The pattern of Cox-2 immunoreactivity was similar in adjacent tissue sections stained with a different anti-Cox-2 antibody from Santa Cruz Biotechnology Inc. (Fig. 4G). Epithelial cell Cox-2 protein expression was prominent in nests of cells at the base of the tumour and within cells invading neighbouring muscularis mucosae (Fig. 4F,G). Epithelial cell Cox-2 expression was driven from the endogenous *Cox-2* gene rather than epithelial cell transgene expression based on the absence of consistent co-localization of lysozyme and Cox-2 proteins in epithelial cells (Fig. 4H–J) and our inability to detect transgenic transcripts in micro-dissected epithelium from transgenic adenomas (data not shown).

By contrast, there was no increase in stromal Cox-2 immunoreactivity in transgenic *cLys-Cox-2* x *Apc* ^*Min/*+^ mouse tumours anywhere in the SI compared with non-transgenic *Apc* ^*Min/*+^ mouse SI tumours (median [inter-quartile range] non-transgenic Cox-2 score 2^1–3^ for n = 27 *versus* transgenic Cox-2 score 1^1–3^ for n = 25; P = 0.29, Mann-Whitney U test). Subgroup analysis of adenomas restricted to proximal, middle or distal thirds of the SI similarly demonstrated no significant difference in stromal Cox-2 scores between transgenic and non-transgenic *Apc* ^*Min/*+^ mouse adenomas (data not shown). There was also no evidence of adenomatous epithelial cell Cox-2 localisation in transgenic *cLys-Cox-2* x *Apc* ^*Min/*+^ mouse SI adenomas, unlike counterpart colonic tumours.

### Increased macrophage Cox-2 in *cLys-Cox-2* x *Apc*^*Min/*+^ mouse colonic adenomas drives nuclear β-catenin localization via paracrine macrophage-epithelial cell signalling

**Figure 5:**
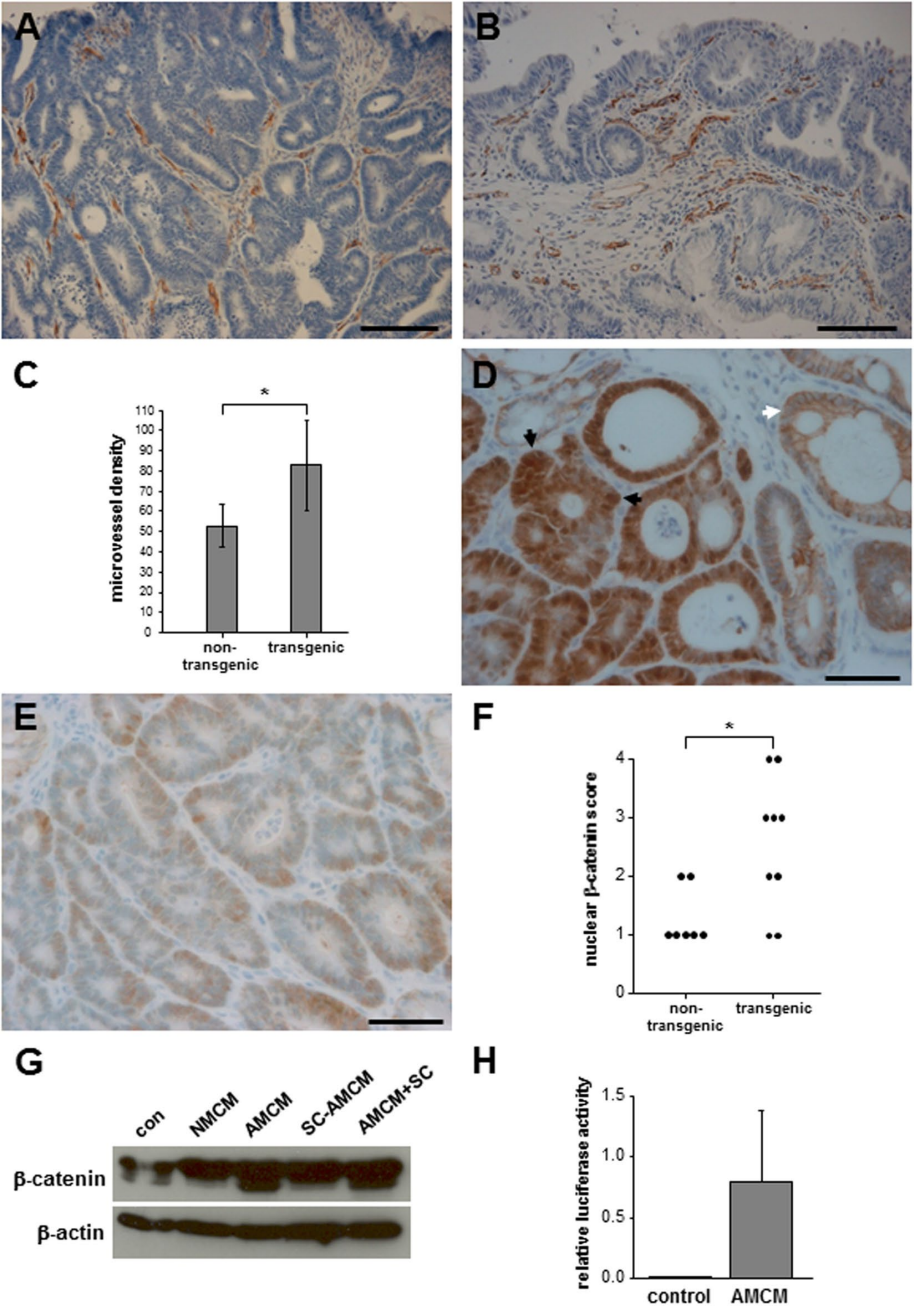
Transgenic *cLys-Cox-2* x *Apc* ^*Min/*+^ mouse colonic tumours have increased tumour-associated angiogenesis and nuclear β-catenin localisation in dysplastic epithelial cells. (**A**) Immunohistochemistry for mouse CD31 demonstrating capillary microvessels in a non-transgenic *Apc* ^*Min/*+^ mouse colonic tumour. Size bar = 50 μm. (**B**) CD31-positive endothelial cells in microvessels in a transgenic *cLys-Cox-2* x *Apc* ^*Min/*+^ mouse colonic tumour. Size bar = 50 μm. (**C**) Microvessel density (number of CD31-positive foci per ‘hotspot’ 100 x microscopic field) in *Apc* ^*Min/*+^ mouse colonic tumours. Bars represent the mean and SEM of the MVD for non-transgenic *Apc* ^*Min/*+^ mouse tumours (n = 8) and transgenic *cLys-Cox-2* x *Apc* ^*Min/*+^ mouse tumours (n = 6). *P = 0.25; Student’s t test). (**D**) Immunohistochemistry for β-catenin on a transgenic *cLys-Cox-2* x *Apc* ^*Min/*+^ mouse colonic tumour demonstrating strong nuclear and cytoplasmic β-catenin immunoreactivity (score 4) in dysplastic epithelial cells (black arrows) compared with the membranous distribution of β-catenin and absence of nuclear staining in neighbouring non-neoplastic epithelium (white arrow). Size bar = 50 μm. (**E**) Immunohistochemistry for β-catenin on a non-transgenic *Apc* ^*Min/*+^ mouse colonic tumour demonstrating patchy, weak nuclear β-catenin staining (score 1) in dysplastic epithelial cells. Size bar = 50 μm. (**F**) Nuclear β-catenin scores of non-transgenic *Apc* ^*Min/*+^ (n = 7) and transgenic *cLys-Cox-2* x *Apc* ^*Min/*+^ (n = 9) mouse colonic tumours. P = 0.03, Mann-Whitney U test). (**G**) Western blot analysis of β-catenin protein content of rat IEC-6 epithelial cells. Cells were cultured for 12 weeks in the presence of control medium alone (con), non-activated macrophage-conditioned medium (NMCM), activated macrophage-conditioned medium (AMCM), activated macrophage-conditioned medium (AMCM) produced in the presence of 1 μM SC-236 (SC-AMCM) or activated macrophage-conditioned medium (AMCM), to which 1 μM SC-236 was added after production (AMCM ^+^ SC). The β-catenin doublet was 90-92 kDa in size. β-actin (42 kDa) was used as a loading control. The images of the blots are cropped but no bands have been omitted by editing. (**H**) TOPflash activity in control IEC-6 cells (con) or IEC-6 cells cultured in the presence of AMCM for 12 weeks. Data are TOPflash firefly luciferase values normalised to Renilla luciferase activity. No FOPflash luciferase activity was detectable (data not shown). Columns and bars represent the mean and SEM of triplicate values.

We have previously reported that the presence of stromal Cox-2-positive macrophages in human colorectal adenomas is associated with an increased microvessel density (MVD) indicative of increased angiogenesis, which could account for accelerated intestinal tumorigenesis in *cLys-Cox-2* x *Apc* ^*Min/*+^ mice^11, 27^. Therefore, we measured the CD31-positive MVD of transgenic and non-transgenic *Apc* ^*Min/*+^ mouse colonic adenomas (Fig. 5A,B). Increased MVD was observed in transgenic colonic tumours but the difference from non-transgenic tumours did not reach statistical significance (Fig. 5C).

Alternatively, direct paracrine signalling between macrophages and neighbouring epithelial cells could explain the increased colonic tumour multiplicity and progression observed in transgenic *cLys-Cox-2* x *Apc* ^*Min/*+^ mice. Castellone *et al*.^28^ have reported that PGE_2_ can stimulate β-catenin-related transcription in human CRC cells lacking *Adenomatous Polyposis Coli* function. It is also established that dysplastic epithelial cells in *Apc* ^*Min/*+^ mouse adenomas exhibit increased levels and nuclear localization of β-catenin, following loss of the second *Apc* allele^29^. Therefore, we compared localization of β-catenin protein in transgenic and non-transgenic colonic tumours (Fig. 5D–F). β-catenin immunoreactivity was increased and localized to the nucleus in epithelial cells of *Apc* ^*Min/*+^ mouse colorectal tumours compared with neighbouring non-neoplastic mucosa (Fig. 5D). Moreover, nuclear localization of β-catenin was increased significantly (P = 0.03) in transgenic *cLys-Cox-2* x *Apc* ^*Min/*+^ mouse tumours compared with non-transgenic *Apc* ^*Min/*+^ mouse tumours (Fig. 5F) indicative of increased β-catenin-related transcription in transgenic tumours.

We have previously described Cox-2-mediated pro-tumorigenic macrophage-intestinal epithelial cell signalling in a co-culture model using RAW264.7 mouse macrophages and rat IEC-6 cells^16^. Therefore, we tested whether macrophage Cox-2 activity increased β-catenin levels and β-catenin-related transcription in epithelial cells, in this *in vitro* model. Cells cultured in the presence of Cox-2-positive activated macrophage-conditioned medium contained higher levels of β-catenin protein (particularly the smaller 90 kDa product) compared with control cells (Fig. 5G). Importantly, if the activated macrophage-conditioned medium was produced in the presence of the selective Cox-2 inhibitor SC-236, there was less induction of β-catenin in epithelial cells (Fig. 5G). However, addition of SC-236 after the medium had been conditioned did not have any effect on β-catenin levels, thereby ruling out a direct effect of SC-236 on epithelial cells (Fig. 5G). We also observed increased β-catenin-related transcriptional activity (measured by the β-catenin/T cell factor-reporter TOPflash) in epithelial cells cultured in the presence of activated macrophage-conditioned medium, which is consistent with the increase in β-catenin protein levels detected by Western blot analysis.

Overall, we have demonstrated that nuclear localization of β-catenin (a tissue biomarker of β-catenin-related transcription) is increased in transgenic Cox-2-over-expressing *cLys-Cox-2* x *Apc* ^*Min/*+^ mouse tumours and that paracrine macrophage Cox-2 activity directs increased β-catenin levels and transcriptional activity in epithelial cells.

## Discussion

Our transgenic, macrophage-specific Cox-2 over-expression model provides the first direct evidence that increased stromal macrophage Cox-2 can drive *Apc* ^*Min/*+^ mouse tumour progression in the colorectum, analogous to human colorectal polyp growth and malignant progression. This confirms stromal macrophage Cox-2 as a tumour-specific target for secondary chemoprevention of CRC. Previously, ‘knockout’ studies have implicated a role for Cox-2 in the early stages of intestinal tumorigenesis^1, 2^. Despite one of the studies confirming that expression of *Cox-2* in mouse mutant *Apc* intestinal adenomas was restricted to stromal rather than epithelial cells^1^, these reports stopped short of providing proof that stromal cell *Cox-2* up-regulation could drive rodent intestinal tumorigenesis. Our data now provide a strong rationale for targeting *COX-2* expression in tumour-associated macrophages as a CRC chemoprevention strategy that is unlikely to share the cardiovascular toxicity associated with systemic pharmacological COX-2 inhibition^8^. For example, tumour-associated macrophages are now recognised as a target for anti-cancer nanotherapy^30^. Stromal macrophages also secrete other pro-tumorigenic mediators including WNTs and multiple chemokines/cytokines^31, 32^. However, it should be noted that other cell types contribute to stromal COX-2 expression in intestinal adenomas, at least in the mouse, namely fibroblasts and endothelial cells^12^.

Macrophage Cox-2-dependent tumour growth and progression in *Apc* ^*Min/*+^ mice was restricted to the colon and was not observed in the SI. This may be due to the larger stromal inflammatory infiltrate observed in large colonic tumours^33^, thus driving an increased transgenic Cox-2-dependent paracrine signal. Our data are compatible with those of Cherukuri and colleagues, who reported that myeloid cell-specific *Cox-2* deletion in a *Cox-2* ^*fl*^ *°* ^*x/fl*^ *°* ^*x*^ x *LysM* ^*Cre/*+^ mouse model did not alter SI tumour number or size^34^. In that study, the small number of colonic tumours precluded specific analysis of the effect of myeloid cell-specific *Cox-2* deletion in the colon^34^.

Pharmacological inhibition of stromal macrophage COX-2 expression is dependent on understanding the mechanistic basis of macrophage infiltration and *COX-2* up-regulation in intestinal adenomas. COX-2-positive tumour-associated macrophages in human (and mouse) intestinal adenomas exhibit a phenotype that is not entirely compatible with either classical (M1) macrophage activation or alternatively activated macrophages (M2). For example, Cox-2-positive tumour-associated macrophages in *Apc* ^*Min/*+^ mouse adenomas do not express inducible *nitric oxide synthase (Nos) 2* ^35^, which would be expected in classically activated M1 macrophages^36, 37^. Instead, F4/80-positive macrophages in *Apc* ^*Min/*+^ mouse tumours express arginase I, a recognised M2 marker^15^. Moreover, COX-2-positive macrophages in human colorectal adenomas contain activated nuclear factor κB^38^, which has recently been implicated in maintenance of an M2 phenotype of tumour-associated macrophages^39^. Mosser and Edwards^36^ have proposed a classification of macrophage populations based on different activities (host defence, wound healing or immune regulation), which can co-exist such that macrophages can exhibit features at either end of the macrophage phenotype spectrum with inherent plasticity. We hypothesize that Cox-2-positive, Nos2-negative, NF*κ*B-positive macrophages in intestinal adenomas likely exhibit a ‘regulatory’ phenotype. Interestingly, pharmacological Cox-2 inhibition in the *Apc* ^*Min/*+^ mouse has been reported to switch tumour-associated macrophages from an M2 to M1 phenotype^33^.

The micro-environmental stimulus for macrophage Cox-2 up-regulation remains unknown. Predominant localization of Cox-2-positive cells below eroded surface epithelium in intestinal adenomas suggests that exposure to luminal antigen may explain *Cox-2* up-regulation in macrophages^40^. However, we have previously been unable to demonstrate an intestinal permeability defect in *Apc* ^*Min/*+^ mouse intestine^24^. Intracellular CRC-associated *E. coli* has been demonstrated to drive persistent COX-2 expression in human THP-1 macrophages^41^. Alternative suggested stimulatory signals include mucins produced by neighbouring epithelial cells^42^, secondary bile acids^43^ and direct epithelial-to-stromal cytokine/chemokine signalling eg. by monocyte chemoattractant protein 1^44^. Interplay with other immune cell types, including regulatory T cells, is also likely to modulate the macrophage phenotype^32, 45^, although this has not been studied in detail in colorectal adenomas, as opposed to CRCs.

Although Cox-2 expression is restricted to the stromal cell compartment in *Apc* ^*Min/*+^ mouse intestinal tumours^9, 12, 13, 15^ and is predominantly localised to stromal cells in human colorectal adenomas [10–11,14, 46–47), COX-2 is strongly expressed by malignant epithelial cells in CRCs suggesting a stage-specific switch to epithelial cell COX-2 expression^11, 46^. This is the first report of epithelial cell Cox-2 localization in *Apc* ^*Min/*+^ mouse colonic adenomas. We propose that epithelial cell Cox-2-expression in our *cLys-Cox-2* x *Apc* ^*Min/*+^ mouse model is analogous to the switch to epithelial cell COX-2 protein expression in human colorectal adenomas that exhibit ‘advanced’ features (increased size, high-grade dysplasia, villous architecture) associated with tumour progression to cancer^11, 47, 48^. Consistent with stage-specific Cox-2 expression during colorectal carcinogenesis, Al-Salihi and colleagues have previously reported that intestinal epithelial cell Cox-2 drives progression, but not initiation, of azoxymethane-induced colonic tumours^49^. Pro-tumorigenic activity of COX-2 in different tumour cell compartments suggests that COX-2 likely has autocrine/paracrine effects independent of cell-specific expression.

Severe dysplasia and local invasion of intestinal adenomas in the *Apc* ^*Min/*+^ mouse model is rarely seen^18^. The *cLys-Cox-2* x *Apc* ^*Min/*+^ mouse may find use as a model of carcinoma-*in-situ*, also known as malignant colorectal polyp, which has become a clinically important lesion, especially with the advent of colonoscopic screening.

We report that tumour-associated macrophage Cox-2 over-expression was associated with increased nuclear β-catenin localization in *Apc* ^*Min/*+^ mouse colonic adenomas and that paracrine Cox-2-dependent signalling from macrophages increases catenin-related transcription in intestinal epithelial cells. Exogenous PGE_2_ has been demonstrated to activate Wnt signalling in human CRC cells *in vitro* via EP2 receptor-dependent activation of PI3K/AKT and direct inhibition of glycogen synthase kinase 3β^28^. Future work will determine whether PGE_2_-EP2 receptor signalling (or that of other EP receptor family members, particularly EP4) in epithelial cells, as well as the stromal cell compartment^15^, explains increased colonic tumorigenesis in *cLys-Cox-2* x *Apc* ^*Min/*+^ mice. In another *in vitro* model, Kaler and colleagues have reported that human THP-1 monocyte-conditioned medium induces catenin-related transcription in HCT116 human CRC cells^50^. It has been proposed that a further increase in β-catenin levels and nuclear translocation of β-catenin, in addition to underlying Wnt signalling dysregulation secondary to *APC* loss of function, driven by stromal cell signalling promotes malignant tumour growth and metastatic behaviour^51^. However, there is not a precise correlation between tumour phenotype and nuclear β-catenin and further work is necessary to delineate paracrine effectors of WNT signal dysregulation in intestinal epithelial cells^52^. Our data suggest that a paracrine COX-2-mediated signal drives tumour progression in cells that have already lost *Apc* function at earlier stages of colorectal carcinogenesis relevant to primary and secondary prevention.

Predominant stromal cell COX-2 localisation during the pre-malignant phase followed by neoplastic epithelial cell COX-2 expression during malignant progression has been observed in other parts of the gastrointestinal tract, in which chronic inflammation predisposes to carcinogenesis, such as reflux oesophagitis/Barrett’s oesophagus^53^ and *Helicobacter pylori*-associated chronic gastritis^54^. Therefore, our transgenic *cLys-Cox-2* mouse model of macrophage Cox-2 expression may be a useful experimental tool for investigation of tumour progression at early stages of carcinogenesis in other parts of the gastrointestinal tract.

## Materials and Methods

### Animals

All experiments were were undertaken with UK Home Office (HO) approval, in accordance with HO guidelines and Institutional policies. Animals were housed in a specific pathogen-free environment.

#### Generation of cLys-Cox-2 transgenic mice

A 5728 bp product consisting of the complete open reading frame of mouse *Cox-2* with 68 bp of 5′-UTR and 239 bp of 3′-UTR was generated by PCR (Extensor Long PCR enzyme mix [ABgene®, Thermo Fisher Scientific, Epsom, UK]) from mouse genomic DNA using primers incorporating *Sal I* (sense) and *Mlu I* (antisense) restriction sites (see Table 1). The PCR product was cloned into *pGEM-T Easy* for confirmatory sequencing.

**Table 1:** PCR primer sequences and expected PCR product size.

Primer pair	Sense (5′-3′)	Antisense (5′-3′)	size (bp)
mCox-2 gDNA^*a*^	dACGC*GTCGAC*GAATCTCAGCACTGCATCCTGCC	dCG*ACGCGT*CTTCTGTTATGGAAGATGTTACATG	5728
mCox-2 cDNA	dTCAAAAGAAGTGCTGGAAAAGGTT	dTCTACCTGAGTATCTTTGACTGTG	296
cLysozyme cDNA	dGATCGTCAGCGATGGAAACGGC	dCTCACAGCCGGCAGCCTCTGAT	101
cβ-actin	dAGATGACACAGATCATGTTT	dTCCACATCACACTTCATGAT	511
cLys-Cox-2 gDNA	dCCACCTGCCACTGAATGGCT	dCGGAAGAGCATCGCAGAGGTG	602
cLys-Cox-2 cDNA	dTCAAAAGGCGTTCAACTGAGC	dCTCACAGCCGGCAGCCTCTGAT	351
real-time mCox-1 gDNA	dTGGAGATGACGGGTCTGTCTTAG	dACTTGTCTTCATCAGGAACAAAACTC	—
real-time mCox-2 gDNA	dGAATTTTTTTTCATGTAACATCTTCCATAA	dGGACAAACACCGGAGGAATCT	—

^a^Sense and antisense primers contain *Sal I* and *Mlu I* recognition sites respectively (italicized).

m, mouse.

c, chicken.

The vector carrying the *cLys* locus was *pIIIiLysV*_*Sal30*_^20^, which contains the complete 21 kb locus except that there is a 700 bp deletion in intron 2 and replacement of the *cLys* translation start site by a *Sal I* restriction site. The genomic mouse *Cox-2* clone was ligated with *Sal I*/*Mlu I*-digested *pIIIiLysV*_*Sal30*_ followed by transformation of ultra-competent *E. coli* (XL-10 Gold, Agilent Technologies, Stockport, UK). The resulting 26 kb *cLys-Cox-2* construct was linearised with *Sfi I* and *BssH I* giving an 18 kb species minus 5 kb of 3′ flanking sequence, which contains the majority of the adjacent chicken *Gas41* gene and 2.1 kb of backbone vector sequence.

The 18 kb vector was then used for pronuclear injection of fertilised oocytes to generate transgenic founder B6 x CBA animals, which were then screened by transgene-specific (*cLys-Cox-2*) PCR.

*cLys-Cox-2* copy number in B6 c*Lys-Cox-2* transgenic mice was determined by comparison with the corresponding endogenous *Cox-1* gene copy number.

Genomic DNA was extracted from ground tail tissue by adding 0.5 ml TKM buffer (10 mM Tris-HCl pH 7.6, 10 mM KCl, 2 mM EDTA, 4 mM MgCl_2_) including 1.25% (v/v) NP-40, centrifugation at 3000 *g* for 5 minutes, followed by re-suspension in 0.4 ml TKM buffer plus 0.75% (v/v) SDS and 20% (v/v) Chelex resin (Bio-Rad Laboratories Ltd., Hemel Hempstead, UK). After incubation at 55 °C for 30 minutes, 0.2 ml of 6 M NaCl was added and the solution was centrifuged at 16000 *g* for 10 minutes. Propan-2-ol (0.35 ml) was added to 0.5 ml of the resulting supernatant and left at −20 °C overnight. DNA was pelleted and washed with 70% (v/v) ethanol by centrifugation at 16000 *g* for 10 minutes and then re-suspended in 25 μl TE buffer at 50°C for 60 minutes.

SYBR-Green™ real-time PCR using Cox-2 (1 μmol/μl) and Cox-1 (500 nmol/μl) primers (see Table 1) was performed as described^55^ using an ABI7700 machine (Thermo Fisher Scientific).

A whole genome library with a typical insert size of approximately 200 bp was generated using NEBNext® Ultra™ DNA library prep (New England Biolabs, Inc., Ipswich, MA). One lane of 100 bp paired-end reads data was generated from the library using a HiSeq 2500 sequencer (Illumina UK, Cambridge, UK) in high volume mode. The sequence data was aligned to sequences consisting of the entire mouse genome (GRCm38) and the chicken lysozyme gene using the BWA aligner^56^. The aligned data were then scanned for read pairs, in which one read was found to align predominantly to the mouse genome and its read mate was found to align primarily to chicken lysozyme sequence. From these reads, a contig of those mapping to the mouse genome was created and possible integration sites for the transgene were found by identifying sequences in the mouse genome that were homologous to the contig consensus sequence.

#### Apc^Min/+^ mouse model of familial adenomatous polyposis

B6 *Apc* ^*Min/*+^ mice from our established colony were bred with B6 c*Lys-Cox-2* mice in order to produce transgenic homozygous c*Lys-Cox-2* x *Apc* ^*Min/*+^ mice and non-transgenic *Apc* ^*Min/*+^ control animals. Genotyping for the *Apc* ^*Min*^ allele was performed as described^35^. Animals were provided with AIN-76 chow (Thermo Fisher Scientific) and drinking water *ad libitum*. *Apc* ^*Min/*+^ mice were sacrificed by cervical dislocation between 100–110 days of age.

### Intestinal phenotype analysis

The number and size of intestinal tumours were measured in whole specimens blinded to genotype using a dissecting microscope as described previously^35^. Proximal, middle and distal thirds of the SI and colon were ‘swiss-rolled’ and fixed in 4% paraformaldehyde (w/v) before embedding in paraffin. Separate tumour and non-neoplastic mucosal samples were OCT-embedded and snap-frozen in liquid N_2_. Samples of spleen, liver, kidney, brain and lung were also collected at the same time.

### Immunohistochemistry and immunofluorescence

Routine histological analysis was performed on 4 μm paraformaldehyde-fixed, paraffin-embedded sections stained with haematoxylin and eosin (H&E).

Immunohistochemistry for Cox-2 using affinity-purified rabbit anti-serum (160106, Cayman Chemical Co., Ann Arbor, MI) was performed as described^9^. Cox-2 immunohistochemistry was also performed using a goat polyclonal anti-Cox-2 antibody (sc-1745, Santa Cruz Biotechnology, Inc., Dallas, TX), which was either indirectly labelled with biotinylated anti-goat immunoglobulin, for visualisation with a biotinylated rabbit anti-rat secondary antibody (DAKO UK Ltd., Ely, UK) and Vectorstain® ABC (Vector Laboratories, Inc.), or directly conjugated with AlexaFluor® 488 (Thermo Fisher Scientific)^57^. Tumour stromal cell Cox-2 immunoreactivity was scored 0–4 based on intensity and distribution in superficial areas of tumours below the luminal surface (0; no staining: 1; weak, patchy staining: 2; moderate staining with some continuity: 3, intense, continuous staining in a distinct area: 4; intense staining throughout the tumour).

Rat monoclonal F4/80 antibody (clone Cl:A3-1; Serotec, Thermo Fisher Scientific) was visualised using a biotinylated rabbit anti-rat-Ig antibody (Santa Cruz Biotechnology, Inc.) and avidin-AMCA (Vector Laboratories, Inc., Burlingame, CA).

MEC 13.3 rat anti-mouse CD31 antibody (BD Biosciences, Oxford, UK) was used to visualise capillary microvessels for determination of tumour MVD blinded to genotype, as described by our Group previously^58^, using antigen retrieval with 20 μg/ml proteinase K for 25 minutes at 37 °C. Immunoreactivity was visualised with a biotinylated rabbit anti-rat secondary antibody (DAKO UK Ltd.) and Vectorstain® ABC (Vector Laboratories, Inc.), using 3,3′-diaminobenzidine as the substrate.

Immunohistochemistry for β-catenin was performed on fixed tissue sections, which had undergone 800 Watt microwave antigen retrieval for 10 minutes in 10 mM citrate buffer pH 6.0, using rabbit polyclonal anti-human β-catenin antibody (Cell Signaling Technology, Inc., Danvers, MA). Visualisation was achieved using a rabbit EnVision™ kit (DAKO UK Ltd.). The number and intensity of positively-stained epithelial cell nuclei in colorectal tumours was counted semi-quantitatively on a scale of 1–4 in a blinded manner.

### Real-time RT-PCR

Total RNA extraction, reverse transcription and real-time PCR was performed as described^59^ using the ΔCt method, with the mean of three ‘housekeeping’ genes (*PsmB6*, *Gapdh*, β*-actin*) as the comparator C_t_ value. Primers for endogenous mouse *Cox-2* are detailed in Table 1.

### Measurement of mucosal and cell-conditioned medium PGE_2_ levels

Intestinal tissue was homogenised in 1 ml ice-cold Lysis Reagent 1 (Biotrak prostaglandin (PG) E_2_ enzyme-linked immunoassay, GE Healthcare, Amersham, UK) in the presence of 10 µM indomethacin (Sigma-Aldrich Co. Ltd., Dorset, UK,) using a 7 ml glass Dounce Homogeniser. Homogenates were centrifuged at 580 *g* for 5 minutes at 4 °C and the supernatant centrifuged again at 100,000 *g* for 1 hour at 4 °C. The resulting solution was added to an equal volume of Lysis Reagent 2 and stored at −20 °C prior to immunoassay and total protein measurement (*DC* Protein Assay [Bio-Rad Laboratories Ltd.]). PGE_2_ levels are quoted as pg PGE_2_/mg total protein.

### Chicken HD11 and mouse bone marrow-derived macrophage experiments

HD11 pro-macrophage cells^60^ were cultured in Iscove’s-modified Dulbecco’s Medium with Glutamax™ supplemented with 8% (v/v) foetal calf serum, 2% (v/v) chicken serum, 100 U/ml penicillin, 100 U/ml streptomycin and 0.15 μM monothioglycerol (all Life Technologies, Thermo Fisher Scientific). Stable transfection with *cLys-Cox-2* and *pMC1 Neo* was performed by the calcium phosphate method in G418-containing selection medium (700 μg/ml).

Mouse bone marrow-derived macrophages were obtained and cultured as described^35^.

Macrophages were activated with either 5 μg/ml LPS alone for HD11 cells or 1 μg/ml LPS, in combination with 100 U/ml γ-IFN for BMDMs, for 16 hours.

### Macrophage-epithelial cell indirect co-culture

Rat IEC-6 intestinal epithelial cells were cultured in medium conditioned by γIFN/LPS-activated or non-activated RAW264.7 mouse macrophages, which had been cultured in the absence or presence of the selective COX-2 inhibitor SC-236, as described^16^.

### Western blot analysis

Western blot analysis was performed as described^61^ using a 1:5000 dilution of goat polyclonal anti-Cox-2 antibody (Santa Cruz sc-1745) and a 1:20000 dilution of mouse monoclonal anti-β-actin antibody (clone 6F9; Sigma-Aldrich Co. Ltd.) in Tris-buffered saline with 0.1% (v/v) Tween-20 and 1% (w/v) dried skimmed milk. Secondary antibodies were either peroxidase-conjugated (DAKO) for chemiluminescence with ECL Western Blot Substrate (Pierce™, Thermo Fisher Scientific) or AlexaFluor 680- (Thermo Fisher Scientific) and IRDye800-conjugated (Rockland Immunochemicals, Limerick, PA) for measurement of Cox-2 protein levels, which were normalised to the β-actin protein level by near-infrared analysis using an Odyssey® detection system (LI-COR Biotechnology UK Ltd., Cambridge, UK). Western blot analysis of rat IEC-6 β-catenin levels was performed using a 1:5000 dilution of mouse anti-chicken β-catenin antibody (6F9, Sigma-Aldrich) as described^62^.

### Transient transfection of intestinal epithelial cells and dual luciferase reporter assay

Transfection of the β-catenin/T cell factor-reporter construct TOPflash and control construct FOPflash, followed by dual luciferase assay of reporter activity controlled for transfection efficiency, was performed as described^61^.

### Statistical analysis

All analyses were performed using SPSS version 20 and statistical significance was assigned if P < 0.05.

## Electronic supplementary material

Supplementary Information
