# Supplementary material for: Paracrine cyclooxygenase-2 activity by macrophages drives colorectal adenoma progression in the ApcMin/+ mouse model of intestinal tumorigenesis
Source: Sci Rep. 2017 Jul 20;7:6074. doi: 10.1038/s41598-017-06253-5 (PMC5519705; doi:10.1038/s41598-017-06253-5)
Supplement: Supplementary file 1 — Supplementary Information [file 41598_2017_6253_MOESM1_ESM.pdf]

Supplementary Information

Paracrine cyclooxygenase-2 activity by macrophages drives colorectal adenoma progression in the *Apc*$^{Min/+}$ mouse model of intestinal tumorigenesis

Mark A Hull[1]

Richard J Cuthbert[1]

CW Stanley Ko[1]

Daniel J Scott[1]

Elizabeth J Cartwright[1]

Gillian Hawcroft[1]

Sarah L Perry[1]

Nicola Ingram[1]

Ian M Carr[2]

Alexander F Markham[2]

Constanze Bonifer[3]

P Louise Coletta[1]

Section of Molecular Gastroenterology[1] and Section of Translational Medicine[2], Leeds Institute of Biomedical & Clinical Sciences and Section of Experimental Haematology, Leeds Institute of Cancer and Pathology[3], University of Leeds, St James's University Hospital, Leeds LS9 7TF, United Kingdom

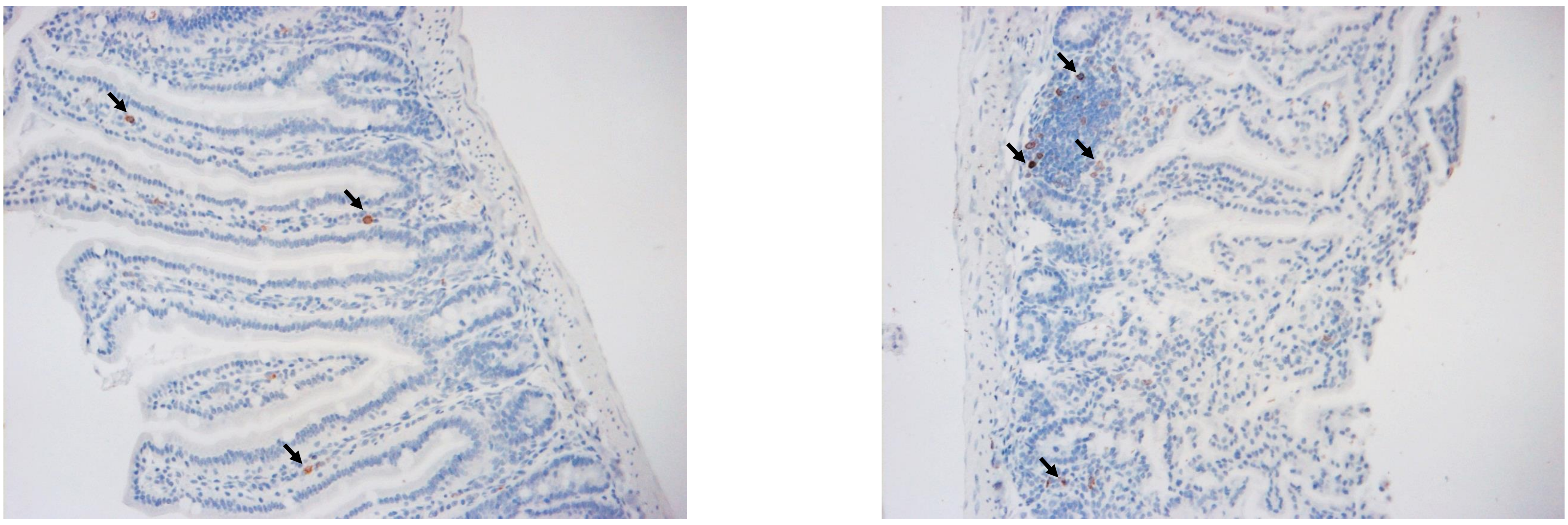

Supplementary Figure 1. Representative photomicrographs of Cox-2 immunohistochemistry on *cLys-Cox-2* x *Apc*$^{Min/+}$ mouse small intestine. Cox-2 immunoreactivity was limited to stromal macrophages and monocytes in lymphoid aggregates (arrows highlight examples). Immunohistochemistry performed with Cayman anti-Cox-2 antibody (see Methods).
